# Supplementary material for: Diversified Rice Farms with Vegetable Plots and Flower Strips Are Associated with Fewer Pesticide Applications in the Philippines
Source: Insects. 2023 Sep 22;14(10):778. doi: 10.3390/insects14100778 (PMC10607731; doi:10.3390/insects14100778)
Supplement: Supplementary file 1 [file insects-14-00778-s001.zip › insects-2595342-supplementary.pdf]

Supplementary Information

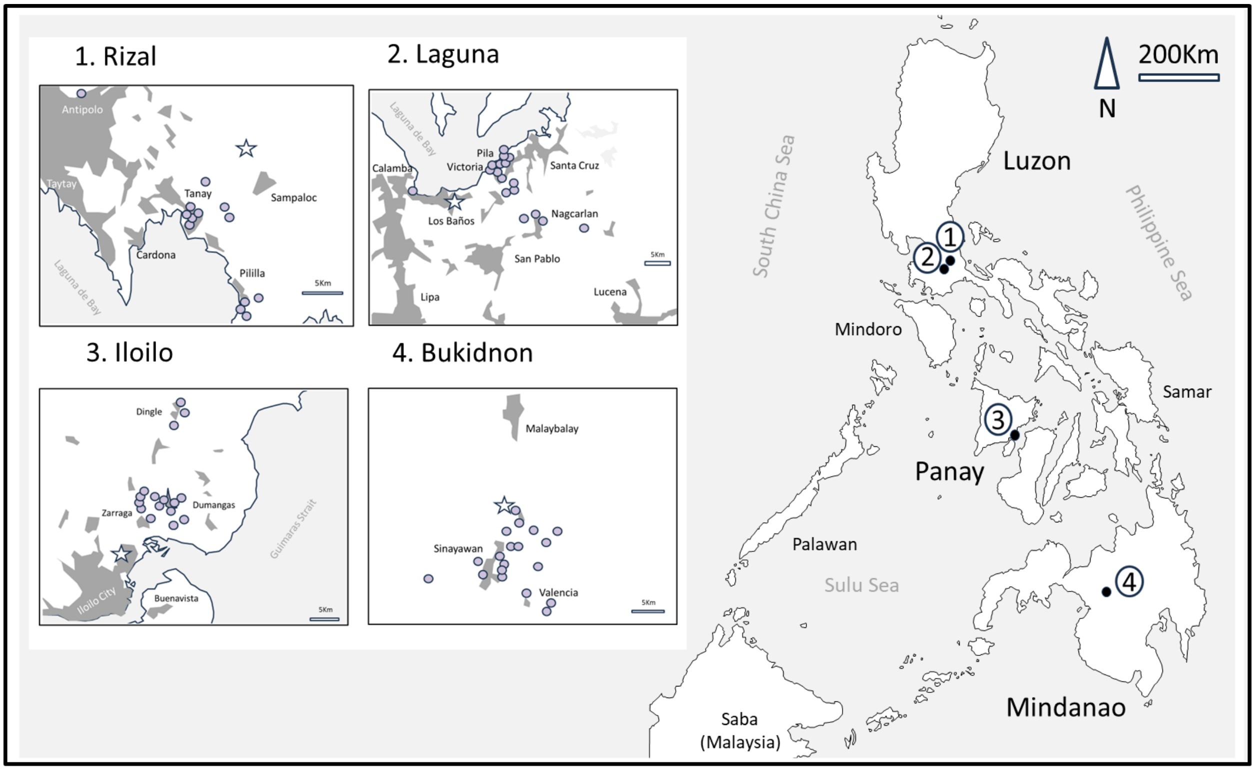

Figure S1. Map of the Philippine Archipelago showing the locations of study regions (numbered in circles), demonstration farms (stars) and farmers' villages (circles). Urbanized areas are indicated in inset maps by dark shading.

Table S1. List of the predictor variables included in the calculation of the DistLM models

Predicting Variable	Type of Variable and Levels
Age	Numerical
Gender	Categorical, 2 levels: male, female
Region	Categorical, 4 levels: Iloilo, Laguna, Bukidnon, Tanay
Education	Categorical, 5 levels: Elementary, Highschool, Undergraduate, Vocational, College graduate, Postgraduate, Self-study
Rice as main crop	Categorical, 2 levels: Yes, No
Other incomes	Categorical, 5 levels: Other crops, Livestock, Retail/shop, Employment, No other income
Experience as a rice farmer	Numerical
Ownership	Categorical, 2 levels: Yes, No
Weekly visits to the farm	Numerical
Belongs to a farmers' organization	Categorical, 2 levels: Yes, No
Rice area	Numerical
Uses biocontrol	Categorical, 2 levels: Yes, No
Harvests ratoon	Categorical, 2 levels: Yes, No
Grows vegetables	Categorical, 2 levels: Yes, No
Diversity of vegetables grown	Numerical
Ecological literacy	Numerical
Wild harvest	Categorical, 2 levels: Yes, No
Flowers planted on bunds	Categorical, 2 levels: Yes, No
Raise ducks	Categorical, 2 levels: Yes, No
Hunts snails in the field	Categorical, 2 levels: Yes, No

Table S2: Profiles of the farmers surveyed at the four sites

Farmer Profiles	Region				DF	Test Statistic1	P-value	Valid Answers
	Laguna2	Rizal2	Iloilo2	Bukidnon2				
1 Age (Years)	55.96 (1.39)b	52.60 (1.60)ab	56.92 (0.89)b	51.04 (1.13)a	3	5.575	0.001	300
2 Gender (% Male)	73.68ab	94.83b	43.75a	60.19ab	3	48.362	<0.001	302
3 Education Attained								
a % Primary (%)	32.14b	23.21ab	7.29a	22.34ab	6	25.823	<0.001	302
b Secondary (%)	39.29a	51.76b	38.54a	32.98a				
c Tertiary (%)	28.57a	25.00a	54.17b	44.68ab				
4 Rice Production as Main Income (%)	85.71	89.29	89.58	84.04	3	1.247	0.65	301
5 Rice-Growing Experience (Years)	26.68 (1.98)	25.29 (1.97)	21.52 (1.53)	23.30 (1.46)	3	1.748	0.157	293
6 Farm Size (ha)	1.52 (0.17)a	1.84 (0.33)a	1.65 (0.13)a	3.02 (0.28)b	3	9.895	<0.001	292
7 Rice Area Owned by Farmer (%)	36.96 (6.59)a	24.89 (5.64)a	64.80 (4.79)b	94.27 (1.94)c	3	42.416	<0.001	292
8 Other Income Generating Activities								
a Produce Other Crops (%)	7.14a	13.79a	18.75a	35.11b	12	33.224	0.001	302
b Farm Livestock or Fish (%)	25.00a	32.76b	23.96a	27.66a				
c Retail (%)	17.86b	15.52b	12.50b	5.32a				
d Other Employment (%)	25.00a	27.58a	32.29a	19.15a				
e No Other Income (%)	25.00a	10.34a	12.50a	12.77a				
9 Land Ownership (% Owing Land)3	30.36a	25.00a	53.13b	72.73b	3	41.153	<0.001	296
10 Farmers Producing Other Crops (%)	44.64a	67.86b	71.88b	75.53b	3	16.856	0.001	302
11 Farm Area Dedicated to Other Crops (ha)4	0.24 (0.07)a	0.44 (0.10)ab	0.42 (0.13)ab	0.74 (0.17)b	3	2.858	0.038	247
12 Farm Area Dedicated to Other Crops (%)4	11.49 (3.11)a	19.00 (3.30)b	13.48 (2.15)ab	15.88 (2.91)ab	3	2.724	0.045	247
13 Visits to Farm (Number per Week)4	5.16 (0.29)ab	5.84 (0.25)b	5.96 (0.19)b	4.37 (0.27)a	3	8.686	<0.001	297

[1]: Test statistics for 1, 5, 6, 7, 11, 12 and 13 are F-value, all others are X2-values. 2: Lowercase letters indicate homogenous groups based on Tukey tests ($P < 0.05$) for F-values and tests of partial homogenization ($P \leq 0.05$). 3: Farmers were categorized as land owners where the majority of their farmed area was self-owned. 4: Data was ranked before analysis.

Table S3: Biotic constraints to rice production during wet (WS) and dry (DS) seasons as indicated by farmers.

Biotic constraint	Laguna		Rizal		Iloilo		Bukidnon		Total number of farmers	
	DS	WS	DS	WS	DS	WS	DS	WS	DS	WS
Rodents/rats	20.41	26.67	14.89	20.51	3.61	2.38	5.13	8.97	24	29
Ricebug (*Leptocorisa* spp.)	12.24	17.78	19.15	20.51	13.25	9.52	14.10	7.69	37	30
Blackbug (*Scotinophara* spp.)	10.20	15.56	2.13		3.61	2.38	23.08	14.10	27	20
Stemborers (*Chilo* spp.; *Scirpophaga* spp.; *Sesamia* sp.)	12.24		8.51	7.69	19.28	14.29	35.90	33.33	54	41
Weeds			4.26	7.69	6.02	4.76	0.00		7	7
Golden apple snail (*Pomacea canaliculata* Lamarck)	22.45	24.44	8.51	15.38	8.43	13.10	0.00	1.28	22	29
Green leafhopper (*Nephotettix* spp.)	4.08	2.22	4.26	5.13	6.02	14.29	3.85	12.82	12	25
Planthoppers (*Niloparvata lugens* Stål; *Sogatella furcifera* (Horvath)			2.13	0.00	14.46	13.10	2.56	5.13	15	15
Blast (*Magnaporther grisea* (T.T. Hebert) M.E. Barr)		2.22		0.00		1.19			0	2
Tungro disease (transmitted by leafhoppers)	6.12	2.22	17.02	10.26	4.82	2.38	2.56	2.56	17	9
Leaf blight (*Xanthomonas oryzae* (Ishiyama) Swings et al)			0.00		2.41	3.57	1.28		4	3
Army worm (*Mythimna* spp.)	12.24		2.13				2.56	1.28	9	1
Birds			8.51	7.69	2.41	2.38			6	5
Fungi		4.44	2.13		1.20	3.57			2	5
Mole cricket (Gryllotalpidae)		2.22			1.20				1	1
leaffolder (*Cnaphalocrocis medinalis* (Guenee))				2.56	1.20	1.19			1	2
ladybeetles (Coccinellidae: Coleoptera)			4.26	2.56					2	1
maggots (*Hydrellia* spp.)						1.19		1.28	0	2
Grasshoppers			2.13						1	0
brownspot (*Cochliobolus miyabeanus*)					1.20				1	0

Caterpillars	1.28	1.28	1	1
Rice grain bug (*Paraeucosmetus pallicornis* (Dallas))	6.41	6.41	5	5
crawfish		1.28	0	1

1: Numbers are percentages of farmers mentioning each constraint as their main (top ranked) problem.

Table S4: Differences between farmers' perception about the most harmful pests, based on region. SIMPER analyses show the pests (in order of importance) contributing most to the dissimilarity between regions, in both seasons.

Season	Pest species	Iloilo vs. Laguna		Iloilo vs. Tanay		Iloilo vs. Bukidnon		Laguna vs. Bukidnon		Laguna vs. Tanay		Bukidnon vs. Tanay	
		Contrib. %	Cum. %	Contrib. %	Cum. %	Contrib. %	Cum. %	Contrib. %	Cum. %	Contrib. %	Cum. %	Contrib. %	Cum. %
Dry Season	Stemborer	13.14 (I)	13.14	13.99 (I)	13.99	15.52 (B)	15.52	15.85 (B)	15.85	13.50 (L)	13.50	16.50 (B)	16.50
	Rice bug	13.00 (I)	26.14	14.23 (T)	28.22	13.61 (B)	29.13	13.92 (B)	29.77	14.70 (T)	28.20	15.02 (T)	31.52
	Black bug	10.33 (L)	36.47	7.09 (T)	35.31	11.52 (B)	40.65	14.21 (B)	43.98	11.80 (L)	40.00	12.78 (B)	44.30
	Rats	9.57 (L)	46.04	10.33 (T)	45.64	6.68 (I)	47.33	9.89 (L)	53.87	12.88 (T)	52.88	10.49 (T)	54.79
	GAS	8.19 (L)	54.23	6.69 (I)	52.33	6.30 (I)	53.63	7.72 (L)	61.59	8.12 (L)	61.00	5.68 (T)	60.47
Wet season	Stemborer	11.28 (I)	11.28	12.28 (I)	12.28	14.60 (B)	14.60	15.02 (B)	15.02	11.36 (T)	11.36	15.32 (B)	15.32
	Rice bug	13.50 (L)	24.78	12.40 (I)	24.68	13.06 (B)	27.66	14.61 (L)	29.63	13.99 (L)	25.35	13.07 (B)	28.39
	Rats	10.70 (L)	35.48	11.05 (T)	35.73	8.63 (B)	36.29	11.94 (L)	41.57	13.67 (L)	39.02	11.94 (T)	40.33
	Black bug	8.57 (L)	44.05	3.80 (T)	39.53	9.51 (B)	45.80	13.16 (B)	54.73	10.06 (L)	49.08	10.73 (B)	51.06
	GAS	10.67 (I)	54.72	10.76 (I)	50.29	9.97 (I)	55.77	10.23 (L)	64.96	11.00 (L)	60.08	9.91 (T)	60.97

The letter in brackets indicate the region in which each pest is more reported ('B' = Bukidnon, 'I' = Iloilo, 'L' = Laguna, 'T' = Tanay). 'Contrib. %' indicates the contribution to dissimilarity between pairs of regions, expressed as a percentage. 'Cum. %' indicates cumulative dissimilarity between pairs of regions, expressed as a percentage. 'GAS' indicates Golden apple snail.

Table S5. The plants grown by the rice farmers as mentioned during the interviews

English Name	Scientific Name
Ladyfinger	*Abelmoschus esculentus* (L.) Moench
Calamus	*Acorus* spp.
Yellow bells	*Allamanda cathartica* L.
Onions	*Allium cepa* L.
Garlic	*Allium sativum* L.
Amaranth	*Amaranthus viridis* L.
Pineapple	*Ananas comosus* (L.) Merr.
Guyabano	*Annona muricata* L.
Jack fruit	*Artocarpus heterophyllus* Lam.
Asparagus	*Asparagus* spp.
Star fruit	*Averrhoa carambola* L.
Malabar spinach	*Basella alba* L.
Bougainvillea	*Bougainvillea* spp.
Mustard	*Brassica juncea* (L.) Czern.
Kohlrabi	*Brassica oleracea* L.
Pichay	*Brassica rapa* L.
Pigeon pea	*Cajanus cajan* (L.) Millsp.
Bell peppers	*Capsicum annuum* L.
Chilies	*Capsicum* spp.
Papaya	*Carica papaya* L.
Chrysanthemum	*Chrysanthemum indicum* L.
Watermelon	*Citrullus lanatus* (Thunb.) Matsum. & Nakai
Calamansi	*Citrus × microcarpa* Bunge
Coconut	*Cocos nucifera* L.
Taro	*Colocasia esculenta* (L.) Schott
Jute	*Corchorus* spp.
Cosmos	*Cosmos* spp.
Melon	*Cucumis melo* L.
Cucumber	*Cucumis sativus* L.
Pumpkin	*Cucurbita moschata* Duchesne
Squash	*Cucurbita* spp.
Lemongrass	*Cymbopogon citratus* (DC.) Stapf
Carrots	*Daucus carota* subsp. *sativus* (Hoffm.) Schübl. & G. Martens
Soybean	*Glycine max* (L.) Merr.
Camia	*Hedychium philippinense* K. Schum.
Sunflower	*Helianthus annuus* L.
Roselle	*Hibiscus sabdariffa* L.
Hibiscus	*Hibiscus syriacus* L.
Water spinach	*Ipomoea aquatica* Forssk.
Sweet potato	*Ipomoea batatas* (L.) Lam.
Lettuce	*Lactuca sativa* L.
Bottle gourd	*Lagenaria siceraria* (Molina) Standl.
Lantana	*Lantana camara* L.
Luffa	*Luffa acutangula* (L.) Roxb.
Cassava	*Manihot esculenta* Crantz
Butterdaisy	*Melampodium divaricatum* L.
Bitter gourd	*Momordica charantia* L.

Moringa	*Moringa oleifera* Lam.
Banana	*Musa acuminata* Colla
Tobacco	*Nicotiana tabacum* L.
Lima bean	Phaseolus lunatus L.
Green beans	*Phaseolus vulgaris* L.
Peas	*Pisum sativum* L.
Winged bean	*Psophocarpus tetragonolobus* (L.) D.C.
Radish	*Raphanus raphanistrum* subsp. s*ativus (L.) Domin*
Sugarcane	*Saccharum officinarum* L.
Chayote squash	*Sechium edule* (Jacq.) Sw.
Tomato	*Solanum lycopersicum* L.
Eggplant	*Solanum melongena* L.
Marigolds	*Tagetes erecta* L.
Spinach	*Talinum fruticosum* (L.) Juss.
Mung bean	*Vigna radiata* (L.) R. Wilczek
Cowpea	*Vigna unguiculata* (L.) Walp.
Stringbean	*Vigna unguiculata* subsp. *sesquipedalis* (L) Verdc.
Maize	*Zea mays* L.
Ginger	*Zingiber officinale* Roscoe
Zinnia	*Zinnia peruviana* (L.) L.
